# Comparative efficacy of low-concentration trisodium citrate and low-concentration heparin for locking central venous hemodialysis catheters: a randomized controlled study

**DOI:** 10.1080/0886022X.2026.2641842

**Published:** 2026-03-12

**Authors:** Pattharawin Pattharanitima, Kwansuphang Wongwatanasanti, Pichaya Tantiyavarong, Aphichat Chatkrailert, Wanna Banchonglaksa, Suthiya Anumas

**Affiliations:** a Division of Nephrology, Department of Medicine, Faculty of Medicine, Thammasat University, Pathum Thani, Thailand; b Division of Clinical Epidemiology, Faculty of Medicine, Thammasat University, Pathum Thani, Thailand; c Nephrology and Dialysis Unit, Thammasat University Hospital, Thammasat University, Pathum Thani, Thailand; d Chulabhorn International College of Medicine, Thammasat University, Pathum Thani, Thailand

**Keywords:** Catheter locking anti-coagulants, central venous catheters, hemodialysis, trisodium citrate (TSC), unfractionated heparin (UFH)

## Abstract

Central venous catheters (CVCs) are used in hemodialysis patients when arteriovenous fistulas (AVFs) or grafts (AVGs) are not feasible. Catheter-locking anticoagulants (CLAs), such as low-concentration trisodium citrate (TSC) and unfractionated heparin (UFH), are used to prevent catheter dysfunction (CD), but comparative data on their safety and efficacy – especially at low concentration remain limited. This study evaluates the efficacy of low-concentration TSC versus low-concentration UFH as CLAs in hemodialysis CVCs. Patients undergoing hemodialysis were randomly allocated to receiving either 5% TSC or UFH 1000 U/mL as CLAs. The primary outcome was the CD rate. Secondary outcomes encompass catheter-related bloodstream infection (CRBSI), exit site infection (ESI), bleeding events, and mortality at 180 days. A total of 204 hemodialysis patients were randomized to receive either 5% trisodium citrate or 1,000 U/mL unfractionated heparin as catheter-locking anticoagulants. The CD rates at 180 days were 1.11 and 1.73 per 1,000 catheters-days for TSC and UFH, respectively, yielding an incidence rate ratio of 1.55 (95% CI: 0.72–3.45), *p* = 0.23. CRBSI and ESI were comparable in both groups. Bleeding and mortality were also not significantly different between the groups. In the sensitivity analysis of tunnel cuffed catheters (TCC), the rate of CD remained not significantly different between the groups; however, the rate of ESI was higher in the UFH group. Both 5% TSC and 1,000 U/mL UFH demonstrated comparable overall efficacy and safety as CLAs. A possible reduction in infectious complications with 5% TSC was observed only in the tunneled cuffed catheter subgroup.

## Introduction

In hemodialysis practice, arteriovenous fistulas (AVFs) and arteriovenous grafts (AVGs) are the preferred vascular access methods, as they are associated with lower rates of infection and vascular complications in comparison to central venous catheters (CVCs) [1,2]. However, a subset of patients may be unsuitable for AVFs or AVGs placement, necessitating the use of CVCs for dialysis access [3]. However, the complications associated with catheter use, particularly catheter dysfunction (CD) and infection, warrant significant concern among patients. CD is characterized by the inability to maintain the prescribed extracorporeal blood flow necessary for effective hemodialysis. CD can have various causes, including catheter kinking, fibrin deposition, or clot formation. To preserve catheter patency between dialysis sessions, catheter locking anticoagulants (CLAs) are routinely employed [4,5]. The predominant options for CLAs are trisodium citrate (TSC) and unfractionated heparin (UFH) [5]. These anticoagulants play a crucial role in mitigating complications related to catheter dysfunction and ensuring the continuous functionality of catheters during the interdialytic intervals.

**Figure 1.**
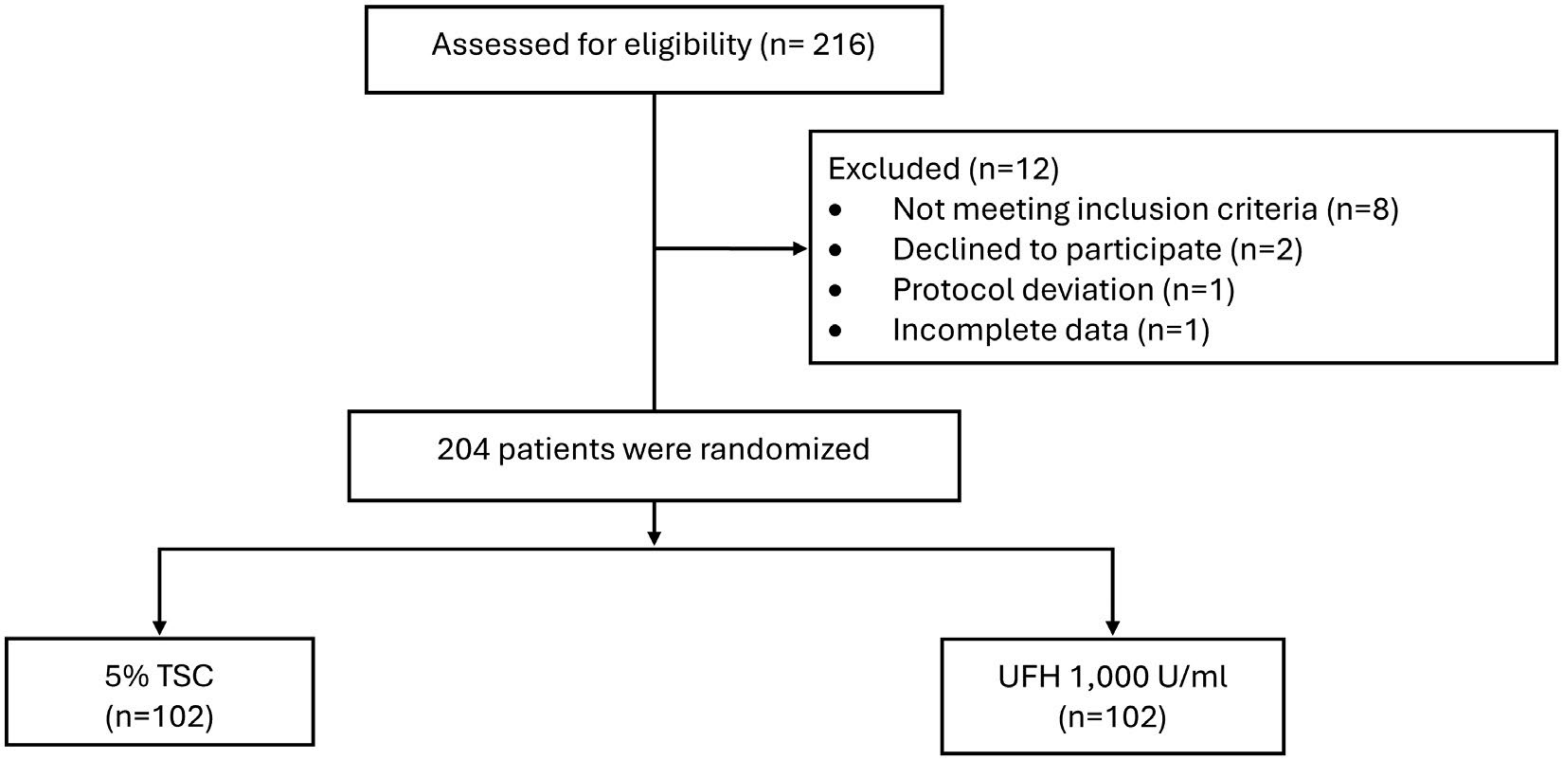
Study flow diagram: Eligibility, randomization, and analysis. **Abbreviation**: TSC; Trisodium citrate, UFH; Unfractionated heparin

UFH, a commonly used anticoagulant due to its rapid onset and proven efficacy, is typically administered at concentrations ranging from 1,000 to 10,000 U/mL. However, it carries a substantial risk of bleeding, particularly at higher concentration [6–8]. Ivan et al. reported that there is no significant difference in cumulative dialysis patency between the use of 1,000 U/mL and 5,000 U/mL concentrations. Although, higher thrombolytic instillations may be necessary with lower concentrations to prevent catheter occlusion [9].

TSC serves as an alternative to heparin, exerting its anticoagulant properties by chelating calcium and disrupting the coagulation pathway. Moreover, TSC demonstrates antimicrobial effects by inhibiting biofilm formation and compromising bacterial cell wall integrity at concentrations above 40% [10]. However, despite the existence of various concentrations, the United States Food and Drug Administration (FDA) issued an urgent advisory in 2000 against the use of 46.7% TSC as a catheter locking agent due to instances of cardiac arrest [11].

Most of studies have reported that 4–5% TSC and UFH 5,000 U/mL have comparable efficacy, with TSC potentially offering additional antimicrobial benefits [12–14]. However, as noted, lower concentration of heparin, such as 1,000 U/mL, may reduce the risk of bleeding complications while maintaining catheter patency similar to that achieved with 5,000 U/mL. This raises the question of why higher concentration of heparin is necessary. Despite this, limited evidence from controlled trial (RCT) has compared the efficacy and safety of low-concentration TSC and low-concentration heparin. Therefore, this RCT aims to investigate the effectiveness and safety of low-concentration TSC and low-concentration UFH as CLAs for CVCs.

## Method

### Study design and participants

This single-center, double-blind randomized controlled trial was conducted at Thammasat University Hospital, Thailand, from June 2023 through June 2024. Eligible participants included individuals over 18 years requiring hemodialysis who had well-positioned non-tunnel cuffed catheters (NTCC) or tunnel cuffed catheters (TCC) expected to be used for more than one week without flow issues, maintaining a blood flow rate (BFR) above 250 mL/min. Exclusion criteria included individuals with hemorrhagic diathesis (e.g. liver failure, disseminated intravascular coagulation, leukemia, vitamin K deficiency, hemophilia, or thrombocytopenia with a platelet count <100,000/μL), hypercoagulable states (e.g. a history of deep vein thrombosis, pulmonary embolism, or antiphospholipid syndrome), hematologic disorders (e.g. paroxysmal nocturnal hemoglobinuria, myeloproliferative neoplasms, polycythemia vera, essential thrombocythemia, or hemolytic anemia), metastatic cancer, current use of oral contraceptives or anticoagulants (e.g. warfarin, UFH, enoxaparin, rivaroxaban, apixaban, or dabigatran), confirmed or suspected heparin-induced thrombocytopenia (HIT), known allergies to heparin, pregnancy, inability to provide essential data, and refusal to participate.

Eligible patients were randomized in blocks of four at a 1:1 ratio to receive either 5% TSC or 1,000 U/mL of UFH as CLAs. Allocation concealment was achieved using opaque, sealed envelopes, and both patients and investigators remained blind to treatment assignments throughout the study. Following each hemodialysis session, each catheter lumen was flushed with 10 mL of normal saline solution (NSS) and subsequently filled with the assigned CLAs, corresponding to the priming volume recommended by the manufacturer for each catheter lumen.

Demographic data of patients including age, sex, hemodialysis (HD) vintage, etiology of end stage kidney disease (ESKD)/acute kidney injury (AKI), underlying medical conditions, current medications, type, duration, and site of CVCs, as well as the history of exit site infection (ESI), catheter-related bloodstream infection (CRBSI), and bleeding events were collected. The laboratory parameters included comprehensive assessments such as complete blood count, coagulogram, renal function tests, electrolyte, calcium, phosphate, and liver function tests.

The primary outcome was the rate of CD, defined as the inability to maintain the prescribed extracorporeal BFR of more than 250 mL/min despite additional NSS flushing and positional change of the patient or the need for recombinant tissue plasminogen activator (rtPA) administration to restore catheter function. Secondary outcomes included rates of CRBSI, ESI, and bleeding events classified as type 3a-5 according to the Bleeding Academic Research Consortium criteria [15]. Mortality was also assessed as a secondary outcome. Diagnostic criteria for CRBSI and ESI were based on the Clinical Practice Guidelines for the Diagnosis and Management of Intravascular Catheter-Related Infections published by the Infectious Diseases Society of America in 2009 [16]. Patients were followed for 180-days post-randomization.

### Statistical analysis

According to a previous study [17–19], the incidence of CD with UFH 5,000 U/mL as a CLAs was reported as 44.8%, 83%, and 12%, with an average of approximately 46.6%. In comparison, the incidence of CD with TSC 4-5% was 40.6%, 67%, and 8.9%, averaging around 38.8%. However, we assume that UFH 1,000 U/mL would result in a higher incidence of CD than 5,000 U/mL, estimated to be around 60%. Based on this assumption, using a power of 80% and a two-tailed alpha of 0.05, the required sample size was estimated at 98 patients per treatment arm. Accounting for a 10% dropout rate, a total of 108 patients were deemed necessary per arm, resulting in a total sample size of 216 patients.

Continuous variables were expressed as mean ± standard deviation (SD) or median and interquartile range (IQR) and compared using unpaired *t*-test or Mann–Whitney U test, as appropriate. Categorical variables were expressed as frequency and percentages and compared using a Chi-square test or Fisher’s exact test. Event rates were reported as the number and percentage of occurrences, incident rates per 1,000 catheters-days, and incidence rate ratio (IRR) in comparison to the UFH group. The analysis of outcomes adhered to an intention-to-treat and statistical significance was determined with a p-value of less than 0.05. The statistical analysis was performed using Stata version 17.0 BE.

### Ethical consideration

The trial protocol was approved by the Human Research Ethics Committee of Thammasat University No 1 (Faculty of Medicine): MTU-EC-IM-0-005/66 and followed the principles of the Declaration of Helsinki and the International Conference on Harmonization Good Clinical Practice guidelines. Written informed consent was obtained from all eligible participants, and the protocol was registered in the Thai Clinical Trials Registry with study number TCTR20231106002. This trial was funded by the Faculty of Medicine, Thammasat University.

## Results

### Baseline characteristics

Among 216 hemodialysis patients, 12 were excluded, resulting in 204 participants who were randomized to either the 5% TSC group or the 1,000 U/mL UFH group. Specifically, 102 patients were assigned to the TSC group and 102 to the UFH group (Figure 1). The NTCC group comprised 49 patients (24.0%), while the TCC group included 155 patients (76.0%). Catheter insertion was indicated for hemodialysis in AKI for 20 patients (9.8%) and for ESKD in 184 patients (90.2%). The baseline characteristics were well balanced, except for the duration of CVC insertion differed between the two groups, with median values of 6.6 months (IQR 0.8 to 17.1) for the TSC group and 3.5 months (IQR 0.2 to 11.7) for the UFH group, indicating a statistically significant difference (*p* = 0.03). Other baseline characteristics were comparable (Table 1).

**Table 1.** Baseline characteristics of patients.

Patients’ demographics	All (*n* = 204)	5% TSC (*n* = 102)	UFH 1,000 U/mL (*n* = 102)	p-value
Age, year; mean (SD)	61.2 (15.8)	62.7 (16.1)	59.8 (15.5)	0.12
Sex: Male; n (%)	88 (43.1)	44 (43.1)	44 (43.1)	1.00
TCC; n (%)	155 (76.0)	83 (81.4)	72 (70.6)	0.10
Comorbidities; n (%)				
Hypertension	193 (94.6)	96 (94.1)	97 (95.1)	1.00
Type 2 diabetes	119 (58.3)	60 (58.8)	59 (57.8)	1.00
Dyslipidemia	160 (78.4)	80 (78.4)	80 (78.4)	1.00
Coronary artery disease	28 (13.7)	13 (12.8)	15 (14.7)	0.84
Current Anti-platelet; n (%)				
Aspirin	49 (24.0)	24 (23.5)	25 (24.5)	1.00
Clopidogrel	7 (3.4)	2 (2.0)	5 (4.9)	0.45
Aspirin and Clopidogrel	18 (8.8)	11 (10.8)	7 (6.9)	0.46
Reasons for CVCs insertion; n (%)				0.81
HD in AKI	20 (9.8)	11 (10.8)	9 (8.8)	
HD in ESKD	184 (90.2)	91 (89.2)	93 (91.2)	
Site of CVCs; n (%)				0.56
Internal jugular vein	172 (84.3)	88 (86.3)	84 (82.4)	
Femoral vein	32 (15.7)	14 (13.7)	18 (17.7)	
Duration of catheter insertion (months); median (IQR)	5.0 (0.4–13.9)	6.6 (0.8–17.1)	3.5 (0.2–11.7)	0.03
Dialysis vintage (months); median (IQR)	8 (1.7–26.6)	8.4 (1.8–33.2)	6.8 (1.3–21.3)	0.45
Study duration (days); median (IQR)	119 (34–180)	151 (40–180)	88 (24–180)	0.21
Previous exit site infection or CRBSI in 1 month; n (%)	1 (0.5)	0	1 (1.0)	1.00
Hb (g/dL); mean (SD)	9.9 (1.9)	9.9 (1.7)	9.8 (2.0)	0.48
Platelet (x10^3^/µL); mean (SD)	232.5 (84.2)	226.7 (82.7)	238.4 (85.6)	0.38
INR; mean (SD)	1.25 (0.37)	1.32 (0.51)	1.20 (0.20)	0.55
Correct calcium (mg/dL); mean (SD)	9.2 (1.00)	9.2 (1.0)	9.3 (1.0)	0.35
Phosphorus (mg/dL); mean (SD)	4.4 (1.8)	4.4 (1.7)	4.4 (1.9)	0.49
Albumin (g/dL); mean (SD)	3.6 (0.7)	3.6 (0.6)	3.5 (0.7)	0.61
TB (mg/dL); median (IQR)	0.5 (0.4–0.6)	0.5 (0.4–0.6)	0.5 (0.4–0.6)	0.63
AST (U/L); median (IQR)	21 (15–35)	21 (16–43)	21 (14–33)	0.31
ALT (U/L); median (IQR)	12 (9–24)	11 (7–24)	15 (9–24)	0.21
ALP (U/L); median (IQR)	91 (69–136)	84 (65–155)	98 (74–133)	0.50

**Abbreviations:** AKI; Acute kidney injury, ALP; Alkaline phosphatase, ALT; Alanine transaminase, AST; Aspartate aminotransferase, CRBSI; Catheter related blood stream infection, CVCs; Central venous catheters, ESKD; End stage kidney disease, Hb: Hemoglobin, HD; Hemodialysis, INR; International normalized ration, TB; total bilirubin, TCC; Tunnel cuffed catheter, TSC; Trisodium citrate, UFH; Unfractionated heparin.

### Catheter dysfunction

At 180 days, the incidence rate of CD was 1.11 per 1,000 catheters-days, corresponding to 13 patients (12.8%) in the TSC group and 1.73 per 1,000 catheters-days, corresponding to 18 patients (17.7%) in the UFH group, resulting in an IRR of 1.55 (95% CI: 0.72–3.45, *p* = 0.23) (Table 2). Based on the definition of CD, the rate of thrombolytic therapy was 0.09 in the TSC group and 0.10 per 1,000 catheters-days in the UFH group, with an IRR of 1.12 (95% CI: 0.01–88.09, *p* = 0.94). The incidence rate of BFR <250 mL/min was 1.11 and 1.73 per 1,000 catheters-days in the TSC and UFH groups, respectively, yielding an IRR of 1.55 (95% CI: 0.72–3.45, *p* = 0.23).

**Table 2.** Primary and secondary outcomes.

Outcomes rate	5% TSC (*n* = 102)	UFH 1,000 U/mL (*n* = 102)	Incidence rate ratio	p-value
**Primary outcome**				
Catheter dysfunction (per 1,000 catheters-days)	1.11	1.73	1.55 (0.72–3.45)	0.23
Catheter dysfunction; n (%)	13 (12.8)	18 (17.7)		0.44
**Secondary outcomes** (per 1,000 catheters-days)				
CRBSI	0.09	0.38	4.49 (0.44–221.07)	0.18
ESI	0.09	0.58	6.73 (0.82–309.73)	0.051
Bleeding episode type 3a-5	0.09	0.10	1.12 (0.01–88.09)	0.94
All-cause mortality	0.51	0.96	1.87 (0.62–6.26)	0.23
Death from infection	0.09	0.38	4.49 (0.44–221.07)	0.18
Death from myocardial infarction	0.17	0	0 (0-5.98)	0.28
Death from arrhythmia	0.09	0.10	1.12 (0.01–88.09)	0.94
Death from stroke	0	0.10	NA	0.47
Death from other causes	0.17	0.38	2.24 (0.32–24.81)	0.38

**Abbreviations:** CRBSI; Catheter related blood stream infection, ESI; Exit site infection, NA; Not applicable, TSC; Trisodium citrate, UFH; Unfractionated heparin.

**Figure 2.**
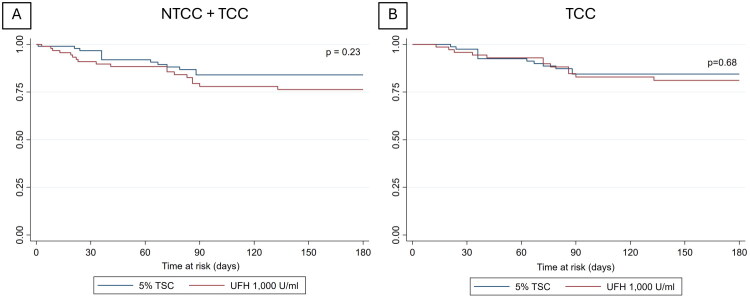
Kaplan-Meier curve for catheter dysfunction. **Abbreviation**: NTCC; Non-tunneled cuffed catheter, TCC; Tunneled cuffed catheter, TSC; Trisodium citrate, UFH; Unfractionated heparin

The Kaplan–Meier survival analysis for catheter dysfunction showed no statistically significant difference between the two groups (*p* = 0.23) (Figure 2(a)).

### Infectious complications

At 180 days, the rates of CRBSI per 1,000 catheters-days were 0.09 in the TSC group and 0.38 in the UFH group, yielding an IRR of 4.49 (95% CI: 0.44–221.07, *p* = 0.18). Similarly, the rates of ESI were 0.09 and 0.58 per 1,000 catheters-days in the TSC and UFH groups, respectively. The corresponding IRR for ESI was 6.73 (95% CI: 0.82–309.73, *p* = 0.051; Table 2).

### Bleeding complication

At 180 days, the incidence rates of bleeding were 0.09 and 0.10 per 1,000 catheters-days in the TSC and UFH groups, respectively, resulting in an IRR of 1.12 (95% CI: 0.01–88.09, p = 0.94; Table 2).

### Mortality

At 180 days, mortality rates were 0.51 and 0.96 per 1,000 catheters-days in the TSC and UFH groups, respectively, resulting in an IRR of 1.87 (95% CI: 0.62–6.26, *p* = 0.23). The causes of death were comparable between the two groups (Table 2).

### Sensitivity analysis

In the sensitivity analysis of TCC, at 180 days, the incidence rate of CD was 1.06 per 1,000 catheters-days in the TSC group and 1.25 per 1,000 catheters-days in the UFH group, resulting in an IRR of 1.18 (95% CI: 0.49–2.88, *p* = 0.68) (Table 3). Based on the definition of CD, the rate of thrombolytic therapy was 0.09 in the TSC group and 0 per 1,000 catheters-days in the UFH group, with an IRR of 0 (95% CI: 0–46.19, *p* = 0.54). The incidence of BFR <250 mL/min was 1.06 and 1.25 per 1,000 catheters-days in the TSC and UFH groups, respectively, yielding an IRR of 1.18 (95% CI: 0.49–2.88, *p* = 0.68). The rate of ESI was higher in the heparin group compared to the 5% TSC group (0 vs. 0.52 per 1,000 catheters-days, *p* = 0.02). However, the rates of CRBSI, bleeding, and mortality were comparable between the two groups.

**Table 3.** Sensitivity analysis of primary and secondary outcomes in patients with TCCs.

Outcomes rate (per 1,000 catheters-days)	5% TSC (*n* = 83)	UFH 1,000 U/mL (*n* = 72)	Incidence rate ratio	p-value
**Primary outcome**				
Catheter dysfunction	1.06	1.25	1.18 (0.49–2.88)	0.68
**Secondary outcomes**				
CRBSI	0	0.21	NA	0.21
ESI	0	0.52	NA	0.02
Bleeding episode type 3a-5	0	0	NA	1.00
All-cause mortality	0.44	0.52	1.18 (0.27–5.15)	0.79
Death from infection	0	0.31	NA	0.10
Death from myocardial infarction	0.18	0	0 (0-6.31)	0.29
Death from arrhythmia	0.09	0	0 (0-46.19)	0.54
Death from stroke	0	0	NA	1.00
Death from other causes	0.18	0.21	1.18 (0.09–16.34)	0.87

**Abbreviations:** CRBSI; Catheter related blood stream infection, ESI; Exit site infection, NA; Not applicable, TSC; Trisodium citrate, UFH; Unfractionated heparin.

The Kaplan–Meier survival analysis for catheter dysfunction showed no statistically significant difference between the two groups (*p* = 0.68) (Figure 2(b)).

## Discussion

This study found no significant difference in the incidence of CD between patients receiving 5% TSC and those receiving 1,000 U/mL UFH. Similarly, the rates of CRBSI, ESI, bleeding, and 180-day mortality did not differ significantly between the two groups. Sensitivity analysis in patients with TCCs showed consistent results for CD; however, the incidence of ESI was higher in the UFH group.

The 2019 KDOQI guidelines suggest that, due to insufficient evidence demonstrating superiority in CVC outcomes, the choice and concentration of TSC or UFH locking solutions should be determined by clinical judgment [3]. However, in clinical practice, concentration reduction may potentially minimize the risk of bleeding and other adverse effects. In comprehensive systematic review and meta-analysis encompassing data from four RCTs involving 370 patients undergoing hemodialysis, the efficacy and safety of UFH at concentrations of 1,000 U/mL (low-concentration) were compared to 5,000 U/mL (high-concentration). This analysis demonstrated no statistically significant differences between the low and high concentrations concerning catheter-related thrombosis, catheter patency, bleeding rates, and occlusion events. Furthermore, the administration of a low-concentration UFH lock significantly reduced activated partial thromboplastin time (aPTT) in comparison to the high-concentration group [8]. These findings substantiate our hypothesis that UFH at a concentration of 1,000 U/mL is efficacious, associated with fewer adverse effects, and therefore more suitable for clinical use. Conversely, the use of TSC as an alternative option may offer potential benefits in reducing the risk of CRBSI and CD. Most studies have primarily focused on comparing the efficacy of 4% citrate with 5,000 U/mL heparin. In a pilot investigation involving 61 hemodialysis patients utilizing TCC, participants were randomly assigned to receive either UFH at 5,000 U/mL or 4% TSC as a locking agent. The findings indicated that the efficacy of 4% TSC and 5,000 U/mL UFH was comparable in preventing CD, ESI, and catheter-associated bacteremia [18]. A recent meta-analysis [20] also reported similar efficacy between citrate and heparin locking solutions for preventing CD. However, the studies included in this outcome analysis were heterogeneous, featuring inconsistent comparison groups – some evaluating low-concentration citrate versus high-concentration heparin, while others compared high-concentration citrate with high-concentration heparin. Although our study utilized low-concentration UFH at 1,000 U/mL compared with low-concentration 5% citrate, the outcomes were consistent with those reported in the aforementioned studies. This further supports the premise that lower concentrations of both heparin and citrate may provide adequate catheter protection while minimizing potential adverse effects. However, the observed incidence rate of catheter dysfunction was lower than anticipated, which may have resulted in limited statistical power to detect between-group differences.

In our study, the TSC group had a longer duration of catheter use compared to the UFH group. As older catheters are generally more susceptible to fibrin sheath formation and subsequent dysfunction, this imbalance would be expected to bias the results toward a higher rate of CD in the TSC group. However, no significant difference in CD rates was observed between groups, suggesting that the efficacy of TSC was comparable to that of UFH despite this unfavorable baseline characteristic. The longer catheter duration in the TSC group may be partly explained by a higher proportion of TCC, which are typically intended for longer-term use. Therefore, we conducted a sensitivity analysis focusing on TCC to evaluate the efficacy of TSC versus UFH. This analysis showed no significant difference in CD between the groups; however, the rate of ESI was significantly higher in the UFH group. A similar trend was observed for CRBSI, although the difference was not statistically significant. This may be attributed to the antibacterial properties of TSC [21]. These findings are consistent with the antimicrobial effects reported by Lok et al. [17], who demonstrated that 4% TSC, compared with 5,000 U/mL UFH, was associated with a significantly lower incidence of CRBSI. These results also align with recent meta-analytic evidence [20] showing that even low-concentration citrate, with or without added antibiotics, reduces CRBSI compared with heparin. In contrast to our results, the meta-analyses reported comparable rates of ESI between citrate and heparin. However, in our study, the antimicrobial effects on CRBSI and ESI were not evident in the overall population (both TCC and NTCCs), possibly because NTCCs had shorter catheter durations, which may have attenuated or masked the antimicrobial advantage of TSC.

Moran et al. [6] has reviewed that appropriate locking solutions for maintaining the patency of CVCs in dialysis include UFH at a concentration of 1,000 U/mL or 4% TSC. However, there is a paucity of studies confirming the comparative efficacy of these catheter locking solutions at low concentration. The results from our investigation substantiate the effectiveness and safety profiles of both 5% TSC and UFH at 1,000 U/mL, indicating no statistically significant differences between the two groups. Therefore, our study indicates that it’s not necessary to argue about which option is better, as both solutions exhibit comparable efficacy without an increase in adverse events – thereby reinforcing the conclusions of Moran’s review. An additional factor to consider is local cost variation; for example, at our institution, 5% TSC is less expensive than 1,000 U/mL UFH, making it a more practical choice in the absence of contraindications. Therefore, given the comparable rates of CD and overall infectious complications between the two CLAs, and the potential added benefit of 5% TSC in reducing infections among patients with TCC, the selection of CLAs should be guided by clinical context, availability, and cost considerations. In routine practice, the required locking volume varies by catheter type, with NTCC typically requiring approximately 1.5 mL per lumen (total of 3 mL) and TCC approximately 2 mL per lumen (total of 4 mL). Using the cost estimates from the present study, the per-procedure cost of 5% TSC was substantially lower than that of UFH at 1,000 units/mL, amounting to 10.9 versus 24.7 Baht (0.79 USD) for temporary catheters and 14.5 versus 33.0 Baht (1.05 USD) for tunneled catheters, respectively. When extrapolated to routine thrice-weekly hemodialysis, these per-session differences may result in meaningful reductions in monthly and annual catheter lock–related expenditures with 5% TSC compared with UFH.

The strength of our investigation lies in its design as an RCT involving a substantial cohort of participants with both NTCC and TCC. This study comprehensively assessed a wide range of clinical outcomes. To our knowledge, it represents the first RCT to compare two CLAs at lower concentrations without the addition of antimicrobial agents. However, there are several limitations to consider. First, we were unable to implement a washout period, primarily because the functional catheters had already been locked with various CLAs to maintain catheter patency. Second, our study was conducted at a single center and included an unequal proportion of NTCC and TCC, as well as differences in baseline catheter vintage between groups. In addition, the observed incidence of catheter dysfunction was substantially lower than the 60% event rate assumed in the sample size calculation, which may have resulted in limited statistical power and an increased risk of a type II error. Finally, the exclusion of patients receiving oral anticoagulants may further limit the generalizability of our findings. Collectively, these limitations warrant cautious interpretation of the results.

## Conclusion

This study demonstrated that 5% TSC and 1,000 U/mL UFH showed comparable overall efficacy in reducing CD, with no significant differences in adverse effects, including mortality. While no statistically significant reduction in infectious complications was observed in the overall cohort, a potential signal toward fewer infectious events was noted in the tunneled cuffed catheter subgroup.

## Data Availability

The data that supports the findings of this study will be made available upon reasonable request to other investigators. All data will be de-identified to ensure the privacy of the study participants, in accordance with ethical guidelines.
